# CS-FCDA: A Compressed Sensing-Based on Fault-Tolerant Data Aggregation in Sensor Networks

**DOI:** 10.3390/s18113749

**Published:** 2018-11-02

**Authors:** Zeyu Sun, Huihui Wang, Baoluo Liu, Chuanfeng Li, Xiaoyan Pan, Yalin Nie

**Affiliations:** 1 School of Computer Science and Engineering, Luoyang Institute of Science and Technology, Luoyang 471023, China; ieliubl@163.com (B.L.); lcflit@163.com (C.L.); xypan0326@163.com (X.P.); Nieyalin111@163.com (Y.N.); 2 Key Laboratory of Intelligent IoT, Luoyang Institute of Science and Technology, Luoyang 471023, China; 3 Department of Engineering, Jacksonville University, Jacksonville, FL 32211, USA; hwang1@ju.edu

**Keywords:** sensor networks, compressed sensing, data aggregation, node clustering

## Abstract

When the nodes in the network are deployed in the target area with an appropriate density, the effective aggregation and transmission of the data gathered in the monitoring area remain to be solved. The existing Compressed Sensing (CS) based on data aggregation schemes are accomplished in a centralized manner and the Sink node achieves the task of data aggregation. However, these existing schemes may suffer from load imbalance and coverage void issues. In order to address these problems, we propose a Compressed Sensing based on Fault-tolerant Correcting Data Aggregation (CS-FCDA) scheme to accurately reconstruct the compressed data. Therefore, the network communication overhead can be greatly reduced while maintaining the quality of the reconstructed data. Meanwhile, we adopt the node clustering mechanism to optimize and balance the network load. It is shown via simulation results, compared with other data aggregation schemes, that the proposed scheme shows obvious improvement in terms of the Fault-tolerant correcting capability and the network energy efficiency of the data reconstruction.

## 1. Introduction

The Wireless Sensor Networks (WSNs) solve the information gathering problem of the physical world for humans and associates the human logical society with the objective physical world [1,2,3]. Therefore, it is generally used in industries, agriculture, medical treatment, environment monitoring, etc. Usually, a great deal of sensor nodes which integrate the functions of sensing, communication, computation, and even storage composes the WSN. It is further established via the multi-hop wireless communication. The target of the WSNs is to monitor the target information within the coverage area and transmit the information to the Sink node or terminal users. Due to the size and cost constraint of the sensor node, its communication, computation, and power supply are greatly restrained [4,5]. Therefore, when the Quality of Service (QoS) of the network is guaranteed, the effective compression of the data is quite necessary. The compression could elucidate the redundant data in the sensing information. Therefore, less communication is required among the nodes to obtain the effective data. Also, the collision is reduced and the network data congestion is thus alleviated while the node energy can be saved and the network lifetime can be prolonged.

In recent years, the compressed sensing theory in the field of signal processing has provided a new solution to this problem [6,7]. The core of the compressed sensing is that, as long as the gathered data is sparse in temporal or spatial domain, we can use some random linear functions to measure the analog signal. As one of the most outstanding breakthrough theories in the area of data compression, the compressed sensing theory has attracted wide attentions in information processing and communications due to its remarkable compression performance and non-adaptive coding as well as the independence of encoding and decoding.

Different from traditional data aggregation techniques, the compressed sensing is suitable for the data aggregation in WSNs for the following reasons [8,9,10]. First of all, it exhibits the remarkable data compression performance and the data gathering and compression can be accomplished simultaneously. Therefore, the total amount of transmitted data is greatly reduced. Secondly, the computational complexity of the encoding process is low and the received vector is obtained simply by linearly projecting the signal through the random measurement matrix. Thirdly, the encoding process and the decoding process are mutually independent and we can adopt different decoding techniques for the same encoding method. The decoding node reconstructs the signal independently into the original data based on the received vector signal. Meanwhile, the reconstruction quality is tolerable against the loss probability of the original data to a certain degree, which guarantees the Fault-tolerant-correcting performance in the data aggregation and reconstruction. According to the analyses above, the usage of the compressed sensing to improve the communication efficiency in the WSNs is advantageous both in theory and in practical techniques. As a result, based on the features of the data transmission among network nodes, we propose a Compressed Sensing-based Fault-tolerant Data Aggregation (CS-FCDA) scheme. Meanwhile, in order to solve the coverage void issue caused by the different node load, we design a low-cost network clustering method which can greatly improve network service property according to the data communication, energy efficiency, load balance and data Fault-tolerant correcting capabilities. As a result, the network lifetime can be prolonged when the QoS is guaranteed.

## 2. Materials and Methods

A basic Compressed Sensing (CS) algorithm was proposed in paper [11] targeted for the compression and reconstruction of the urban traffic data and it was shown that the amount of communication in the network can be significantly reduced. A Distributed and Morphological Operation-based Data Collection Algorithm (DMOA) was proposed in paper [12]. This scheme was based on the non-parametric data construction of the lagged covariance matrix for the reconstruction of the partially lost geological data information. Multiple Target Tracking Algorithm (MTTA) scheme was designed in paper [13] which take the energy consumption of the sensing nodes and the number of sensing data into account while the data was gathered from the nodes and further processed. An asymptotic wavelet data compression algorithm based on effective storage was proposed in papers [14], which determine the data unit of the asymptotic transmission according to the support length of the wavelet function and the available storage capacity of the cluster heads. The sensor nodes for the asymptotic data transmission are chosen according to the spatial correlation. Therefore, the network energy consumption for transmission is reduced while the effective storage is also guaranteed. A compressive sensing function was proposed in paper [15] which based on a proper function base, employs a modified CS technique to improve the data compression quality of the data gathered in the network. There are also other works proposing solutions from other perspectives which could improve the network nodes’ energy efficiency. For example, a clustering based data prediction transmission scheme was proposed in paper [16]. In this scheme, according to mathematical relationship of the expected seamless coverage ratio and the number of required cluster heads, the initial probability for the nodes to contend for the cluster heads are restrained. Meanwhile, the data within the cluster are aggregated using the cluster head nodes and then transmitted to the Sink node. However, this scheme fails to solve the reconstruction issue of the lost data. On the basis of the layered routing algorithm LEACH in paper [17], a clustering which is based on data aggregation algorithm was put forward in paper [18] where cluster heads compress the gathered data through the non-contention method and further performs transmission to the Sink node. Because of the limited energy of the sensor nodes in WSNs, the energy efficiency should be the most important factor considered by the Fault-tolerant-correcting protocols. The energy consumption in WSNs is caused by two reasons, i.e., computation energy consumption and transmitting/receiving energy consumption. However, since the information transmission requires more energy than processing, the protocols employing the processing performance of the sensor nodes would be more efficient in terms of the energy consumption. Therefore, with the given constraint on the sensor nodes, the energy efficiency of the protocol can be improved by the Forward Error Correction (FEC) protocols [19]. Due to the redundant information added in the constructed data packets, the transmission power can be reduced if the encoding is employed in FEC protocols. However, the size of the data packets is increased because of the redundant data, which results in extra energy consumption. This extra energy consumption is caused by the fact that the collision probability of the data tends larger when the transmission time of the data packet increases.

The problem with the schemes above exists from two perspectives. The first one is that most of the data compression and processing is finished by the Sink node in a centralized method which causes the network load imbalance and the coverage void. The second one is that the CS based data gathering and data aggregation schemes only consider the certain characteristic of the service-centered WSNs, such as the data structure, the data correlation or the probability of successful data reconstruction. However, the integral solution based on the overall characteristics remains to be investigated.

## 3. Networks Model

We assume the following characteristics for the network considered in this paper.

(1) All the nodes are isomorphic, i.e., all the nodes share the same sensing radius and communication radius. The nodes employ the directional sensing model in paper [20].
(2) The communication radius of each node is twice as large as the sensing radius of itself. A fully covered network is considered to guarantee the connectivity among the nodes.
(3) We deployed the nodes in the monitoring area uniformly and randomly and the nodes remain time-synchronized.

**Figure 1:**
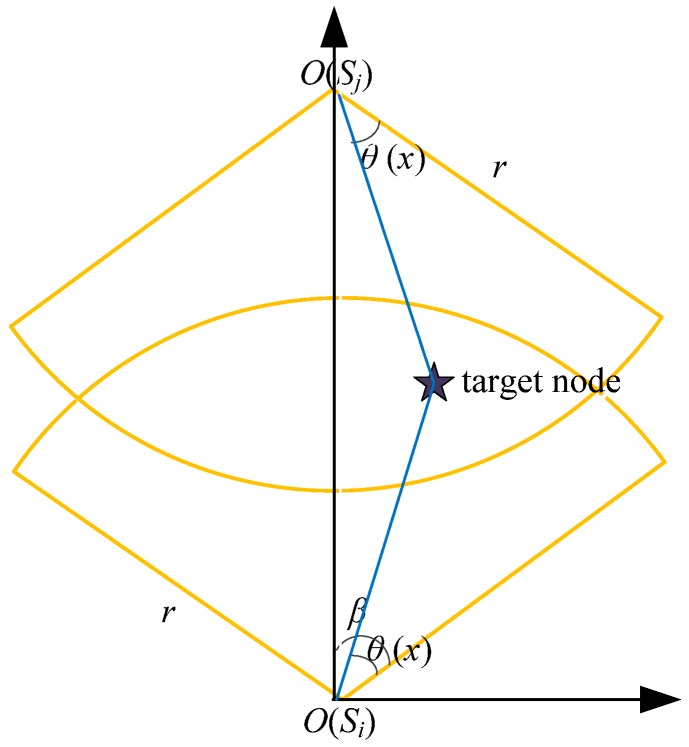
Directional sensing model of the nodes.

**Definition** **1.** **(Node sensing model):** *We define the node sensing model as a four tuple <O,r_s_, α,β>. As we can see from the Figure 1, O and r_s_ represent the position and the sensing radius of the nodes, respectively, while α and β represents the sensing angle and the sensing direction parameter, respectively.*

**Definition** **2.** **(Sensing matrix):***The sensing matrix is used to describe the dynamic environmental information monitored by the sensor network and its mathematical form is described as follows*:

*X* = *x*(*i*, *j*)*_n_*_×*t*_ (1)

*where n and t mean the quantity of the nodes and the quantity of the data gathered at different times.*

**Definition** **3.** **(Reconstruction matrix):** *The reconstruction matrix is an approximate sensing matrix obtained by the data reconstruction algorithm and its mathematical form is as follows:*

*X*′ = *x*′(*i*′, *j*′)*_n_*_×*t*_*.* (2)

**Problem formation:** With a given sensing matrix *X*, we try to compress the data and guarantee that the reconstructed matrix *X*′ is as close to *X* as possible, i.e., the error between *X*′ should be minimized. The mathematical formation is as follows:

(3) $$\underset{x\in R^{N}}{\min}{\left\|X-X\prime \right\|}_{l_{1}}$$

where ||⋅|| is the Euclidean norm between *X*′ and *X*, i.e., $\left\|X\right\|=\sqrt{\sum {}_{i,j}{\left(x\left(x,j\right)\right)}^{2}}$.

**Figure 2:**
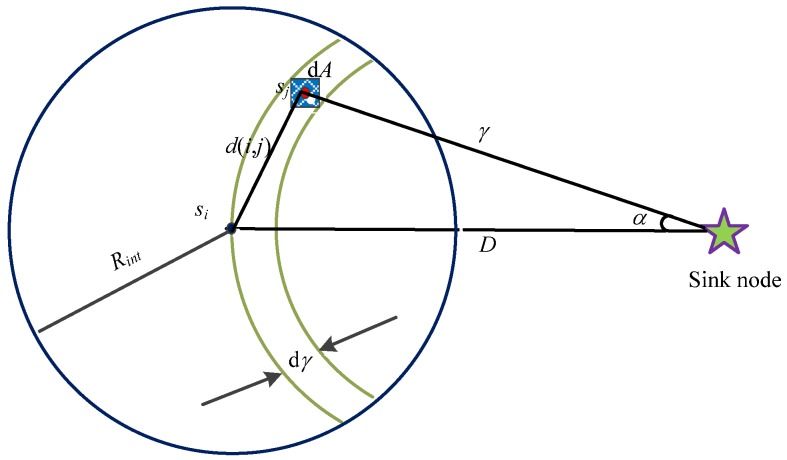
Sensing network model with multi-hop nodes.

The source node *s_i_* transmits the data packets to the Sink node through the path where *s_j_* is chosen for the next hop. The multi-path effect is described by the following logarithmic normal random variable:

PL(*d*) = PL(*d*_0_) + 10*η*(*d*/*d*_0_) + *X_σ_* (4)

where *X_σ_* is random variable obeying the normal distribution *N*(0, *σ*) with mean 0 and variance *σ*^2^. Under this circumstance, when the time *t* → 0, the changing rate of the node transmission range ∆*R_inf_* → 0. Therefore, the transmission range of an arbitrary node is negligible compared with the successful reception probability of one data packet. According to the probability theory, the distance of each individual hop is independent of the change of the parameters. Since different nodes are awakened at different time instants, the network state for each hop is changing, as shown in Figure 2.

The energy consumption is the crucial performance indicator in WSNs. The whole energy which is consumed by the nodes can be defined as the unidirectional outflow energy consumption from node *s_i_* at distance *D.* The definition is given as follows:

*E_flow_*(*D*) = *E*[*E_h_*]*E*[*n_h_*(*D*)]. (5)

In the equation, the expected energy consumption of each single hop is *E*[*E_h_*] while *E*[*n_h_*(*D*)] is the expected number of hops from the aggregation node to node *s_i_* at distance *D.* Then, the end-to-end flow delay of the data packets is defined as:

*T_flow_*(*D*) = *E*[*T_h_*]*E*[*n_h_*(*D*)]. (6)

In the equation, *E*[*T_h_*] is the desired delay of each single hop.

The expected number of hops *E*[*n_h_*(*D*)] can be derived as:

*E*[*n_h_*(*D*)] = [(*D* − *R_int_*)/(*E*[*d_h_*]) + 1]. (7)

In the equation, *D* is the distance of end-to-end and *R_int_* represents the range of the approximate transmission distance while *E*[*d_h_*] is the expected distance of each hop.

### 3.1. Fault-Tolerant-Correcting Scheme of Data Aggregation

In this section, we propose the Compressed Sensing-based Fault-tolerant-correcting Data Aggregation (CS-FCDA). This core of this scheme is to choose the cluster heads in time rounds based on the clustered network. The cluster heads are in charge of data gathering and aggregation. After that the processed data is conveyed to the Sink node, it indeed achieves the reconstruction of the original data.

Take Figure 2 as an example as we performed the analysis and calculation. When ∆*t* → 0, the energy of node *s_j_* will decay to zero, i.e., Δ*E* → 0. Then the node *s_j_* will fall within d*A* = d*γ*d*α* and the Euclidean distance between node *s_i_* and *s_j_* can be expressed as:

(8) $$d\left(i,j\right)=d\left(D,\gamma ,\alpha \right)=\sqrt{\gamma^{2}+D^{2}-2\gamma D\cos\alpha }.$$

In the equation, *D* means the interval distance from node *s_i_* to the aggregation node and *γ* is the interval distance from node *s_j_* to the aggregation node while *α* is the intersection angle between node *s_j_* and the aggregation node.

**Theorem** **1.** *When the conditions above are satisfied, the expected number of hops is:*

(9) $$E\left(d_{h}\right)=\rho \delta \int_{\gamma_{\min}}^{\gamma }\int_{-\alpha_{\gamma }}^{\alpha_{\gamma }}\gamma d_{\left(i,j\right)}Q\left(\frac{\beta }{\sigma }\right)e^{-M\left(\gamma \right)\left(1-p_{k}\left(\gamma \right)\right)}d\alpha d\gamma \;$$

where *ρ* the density of the whole nodes in the network, *δ* is the scale dimension of the parameters and *γ*_min_ is the minimal distance from node *s_i_* to the aggregation node.

**Proof.**  According to the analysis above, when Δ*t* → 0, the energy of the node *s_j_* decays to zero, i.e., Δ*E* → 0. Then, the node *s_j_* will fall within D*a* = d*γ*d*α* where d*A* → 0. Due to the energy consumption of node *s_j_*, the Signal-to-Ratio (SNR) of node *s_j_* exceeds the SNR threshold, i.e., Ψ*_j_* > Ψ*_th_*. Compared with the node *s_j_*, the SNR of an arbitrary node *s_k_* that is much closer to the aggregation node will be smaller than the SNR threshold, i.e., Ψ*_k_* < Ψ*_th_*. So the probability for *s_j_* to be chosen for the next hop is:

d*P*{*N_i_* = *j*} = *P*{*N_A_*(d*γ*) = 1}*P*{Ψ*_j_* > Ψ*_th_*}*P*{*d*_(*j*,*s*)_ ≥ *γ*}. (10)

In the equation, *N_A_*(d*γ*) means the quantity of nodes in the range with size d*A* and interval distance *γ* from the aggregation node. *P*{*N_A_*(d*γ*) = 1} is the probability that only one node exists in the range and *P*{Ψ*_j_* >Ψ*_th_*} is the probability of the event that the received SNR at node *s_j_* from the aggregation node exceeds the SNR threshold Ψ*_th_*. *P*{*d*_(*j*,*s*)_ ≥ *γ*} is the accurate probability which means that the minimal distance from the next hop node *s_j_* to the aggregation node is larger than *γ*. When d*γ* → 0, *N_A_*(d*γ*) = 1 and we can have the following approximation:

*P*{*N_A_*(d*γ*) = 1} ≅ 1 − e^−^*^ρδγ^*^d*γ*d*α*^ (11)

where *ρ* the density of the whole nodes in the network is, *δ* is the scale dimension of the parameters. Since d*γ* → 0 and d*α* → 0, i.e., *ρδγ*d*γ*d*α* → 0, we can simplify the equation above as follows:

*P*{*N_A_*(d*γ*) = 1} ≅ *ρδγ*d*γ*d*α.* (12)

According to Equation (4), when an arbitrary node is at an interval distance which is *d* from the aggregation node, then the received power is:

*P_r_*(*d*) = *P_t_* − PL(*d*_0_) − 10*η*lg(*d*/*d*_0_) + *X_σ._* (13)

In the equation, *P_t_* plays the transmission power, PL(*d*_0_) means the path loss of the reference distance represented by *d*_0_, *η* is for the path loss coefficient and *X_σ_* represents the energy decaying factor with distribution *X_σ_* ~ *N*(0,*σ*). *P_n_* is the power of the noise and the SNR at the aggregation node is:

Ψ(*d*) = *P_r_*(*d*) − *P_n_*. (14)

Denoted by *P*{Ψ*_j_* > Ψ*_th_*}, the probability, the received SNR from the aggregation node at node *s_j_*, exceeds the SNR threshold Ψ*_th_*:

*P*{Ψ*_j_* > Ψ*_th_*} = *P*{*X_σ_* > *β*(*d*_(*i*,*j*), Ψ*th*_)} = *Q*(*β*(*d*_(*i*,*j*), Ψ*th*_)/*σ*) (15)

*β*(*d*_(*i*,*j*),Ψ*th*_) = Ψ*_th_* + *P_n_* = *P_t_* + PL(*d*_0_) + 10*η*lg(*d*(*i*,*j*)/*d*_0_) (16)

(17) $$Q\left(x\right)=\frac{1}{\sqrt{2\pi }}\int_{x}^{\infty }e^{-\left(t^{2}/2\right)}dt$$

where *Q*(*x*) is the *Q*-function of the Gaussian distribution. Since the energy decay follows the lognormal distribution, it represents the probability based on cumulative distribution function of a normal distributed random variable.

*P*{*d*_(*j*,*s*)_ ≥ *γ*} represents the probability which is that the SNR of a node *s_k_* which is closer to the aggregation node than *s_j_* is smaller than the SNR threshold Ψ*_th_*. We denote by *A*(*γ*) the area of the range where an arbitrary node *s_k_* is closer to the aggregation node than node *s_j_*, i.e.,

(18) $$P\left\{d_{\left(i,j\right)}\geq \gamma \right\}=\sum_{i=0}^{\infty }P\left\{N_{A\left(\gamma \right)}=i\right\}p_{k}{\left(\gamma \right)}^{i}=\sum_{i=0}^{\infty }\frac{e^{-M\left(\gamma \right)}M{\left(\gamma \right)}^{i}}{i!}p_{k}{\left(\gamma \right)}^{i}{=e}^{-M\left(\gamma \right)\left(1-p_{k}\left(\gamma \right)\right)}$$

where *A*(*γ*) is the area of the intersection part with radius *R_int_* and *γ*, respectively. Therefore, *N_A_*_(*γ*)_ is the number of nodes which are in *A*(*γ*) and *M*(*γ*) = *ρδA*(*γ*) is the average number of nodes in this area. Additionally, *p_k_*(*γ*) = *P*{Ψ*_k_* ≤ Ψ*_th_*, *k A*(*γ*)} is the probability which is that the node *s_k_* falls in the area *A*(*γ*) and, compared with the threshold, the received SNR is smaller. Therefore, we have:

(19) $$p_{k}\left(\gamma \right)=\int_{\gamma_{\min}}^{\gamma }\int_{-\alpha_{\gamma }}^{\alpha_{\gamma }}\left[1-Q\left(\frac{\beta }{\sigma }\right)\right]\frac{1}{A\left(\gamma \right)}d\alpha d\gamma \;$$

where *γ*_min_ = *D* − *R_int_*.

If we substitute Equations (11) and (13)–(19) into Equation (10), we have the expected number of hops as follows:

(20) $$E\left(d_{h}\right)=\rho \delta \int_{\gamma_{\min}}^{\gamma }\int_{-\alpha_{\gamma }}^{\alpha_{\gamma }}\gamma d_{\left(i,j\right)}Q\left(\frac{\beta }{\sigma }\right)e^{-M\left(\gamma \right)\left(1-p_{k}\left(\gamma \right)\right)}d\alpha d\gamma .$$

This completes the proof. □

### 3.2. Round Mechanism and Network Clustering

**Figure 3:**
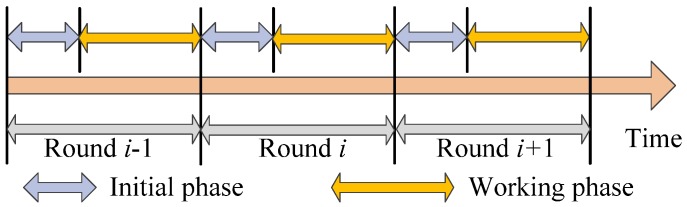
Division of the time rounds.

We employ the time round mechanism here and separate the operation network time into few equal time intervals. Each time interval is defined as one round [21,22,23]. Each round is made up of the initialization period and the work period where the work period is much longer than the initialization period. In the initialization period, the network clustering tasks such as calculating coverage proportion of the node and the election of the cluster head are completed while in the work period, the gathering of the target data and the aggregation task are accomplished. The algorithm is operated in the network in time rounds till the energy of the node is used up. Meanwhile, since no a priori information is required on the network topology, the amount of computation and communication is significantly reduced. The division of the time rounds is illustrated in Figure 3.

First, we divide the network clusters by the coverage area of the nodes. Then, in each cluster, the cluster heads are elected. 

(1) The Calculation of coverage area: In the initialization stage of the time rounds, we divide the monitoring area into some equally large virtual cells (*C*_1_, *C*_2_, …, *C_m_*) according to the sensing and communication radius of the nodes and the requirement of the network coverage task, for example, the regional coverage and the target node coverage. 

We assume that the communication range of an arbitrary node could cover its neighbor nodes so that these two neighboring nodes can transmit information with one another directly [24,25,26]. Each node calculates the coverage area of its own virtual cell and determines its cell membership according to the coverage area. Nodes belonging to the identical cell make a network cluster come into being. For the sake of improving the network property, two factors should be paid much special attention during the clustering process. 

Monitoring target: Each target to be monitored actually should be theoretically covered by one network cluster at the fewest.

Node distribution: The network coverage and connectivity requirement should be satisfied by the node deployment density. Since the cluster size is inversely proportional to the effective coverage area of the nodes, we need to reduce the size of the virtual cells while the effective coverage area of the nodes becomes relatively large. Meanwhile, we should guarantee the successful communication among the neighboring cluster heads.

**Figure 4:**
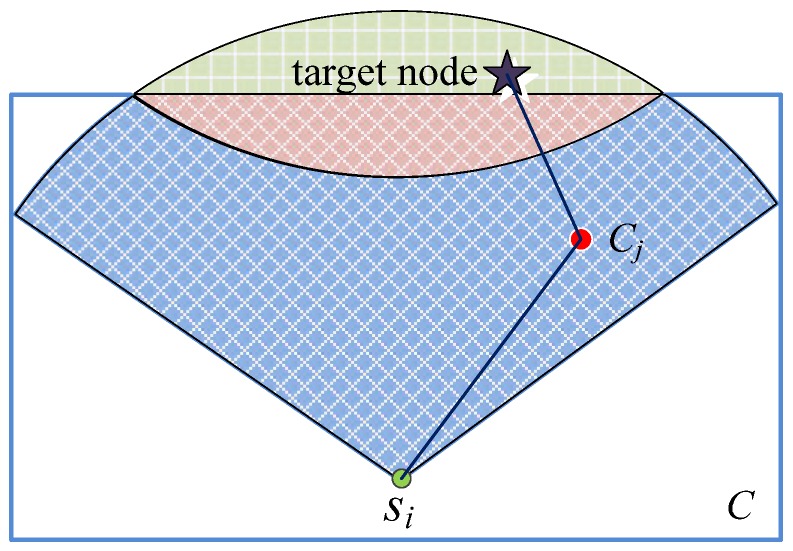
Calculation of network coverage area.

We first calculate the Coverage Area (CA) of an arbitrary node *s_i_* in the virtual cell *C_j_*. After that the node determines its own cell membership according to its largest CA. As shown in Figure 4, the node *s_i_* has the largest coverage area in cell *C_j_*. As a result, node *s_i_* belongs to the virtual cell *C_j_*. This decision is performed at each node in turn and the network clustering is finally finished. 

(2) Election of the Cluster head: Compared with the ordinary cluster members, the cluster heads are in charge of more tasks. Therefore the choice of cluster heads should consider the remaining node energy as well as the node location, i.e., nodes closer to the cell center has higher priority. After the first formation of the network clustering, an arbitrary node close to the cell center is randomly chosen as the cluster head. In subsequent time rounds, the member nodes send a “*vote*” campaign message to the primitive cluster head, which includes the ID of the node, remaining energy and location information. 

We can calculate the probability to choose the node *_Si_* as the cluster head as follows:

(21) $$P_{ch}^{i}=\max\left(\frac{k}{N}\times \frac{E_{r}^{i}}{E_{ini}},\frac{k}{N}\times \frac{E_{\min}}{E_{ini}}\right).$$

In the equation, *N* and *K* respectively represent the number of nodes and clusters. 

The remaining energy of node *s_i_* is represented by *E_r_^i^*. *E*_min_ and *E_ini_* denote the threshold of the minimal energy for the nodes and the initial energy. If *E_r_^i^*, compared with *E*_min_, is smaller, the node will not be elected as the cluster head while the numerical value of *E*_min_ is ensured by the consumed energy for one node to finish one round of data gathering, compression and transmission. 

When the original cluster head receives the “vote” message from the member nodes, it calculates and compares the values of $p_{ch}^{i}$. Then it chooses the node with the largest $p_{ch}^{i}$ as the cluster head, then an “invitation” message to the member nodes is sent by the new cluster head. After the member nodes confirm the reception of the “invitation” message, this round of cluster head election is finished. Within each cluster, the cluster members send the data directly to the cluster heads. However, among different clusters, the cluster nodes aggregate the data within the cluster and that from other cluster heads with the following aggregation algorithm and then compress and transmit the data to the Sink node.

### 3.3. Data Gathering and Compression 

The special signal reconstruction method is employed by the application of CS theories so that a signal Φ can be exactly reconstructed with a sampling rate. The sampling rate is much smaller than the Nyquist criterion. Here, the signal is supposed to exhibit the sparseness feature, i.e., the length-*N* signal *X* is *k*-sparse on a specific orthogonal base. That is, *k* elements of the signal *X* is nonzero and *k* ≪ *N.* We assume that each data element in Φ is independently and identically distributed with normal distribution (0, 1/*N*). The probability density function *p* is given in Equation (18). 

Then we use a matrix of measurement which is uncorrelated with the transformation base to project the high-dimension signal to a low-dimension state. Finally, we can solve the problem which is of optimization and reconstruct the signal which is original with a high probability utilizing a small number of projections.

For the data gathered by the member nodes within the cluster, these data usually exhibit temporal or spatial correlation. In order to impose the sparseness feature on these data, we need to perform further processing. Firstly, we have to elucidate the common parts and maintain the unique parts. Then we compress the unique parts and add the common parts after the reconstruction. Therefore, we can make the reconstruction error reduced and make the data processing speed of the nodes improved while reducing the communication overhead.

Then, we employ the Discrete Cosine Transformation (DCT) to perform the sparseness transformation on the data. The reason for adopting the DCT is that it can concentrate the key information of most signals into the low-frequency part after the transforming. What we can see as follows is the DCT function.

(22) $$f_{k}=\sum_{i=1}^{n}x[i]\cos\left[\frac{\pi }{n}k\left(i+\frac{1}{2}\right)\right]m=1,2,...,n.\;$$

The cluster heads employ the DCT function and transmit the transformed vector to the Sink node. Usually the size of the transformed vector is smaller than 1000 for a rapid reconstruction. Since we adopt the network clustering mechanism, the number of nodes within each cell is smaller than 20 so that the transformed vector is also small in size. As a result, the Sink node could easily reconstruct the original data.

**Theorem** **2.** *For a matrix Φ_s_ = (ξ1,ξ_2_, …, ξ_M_)^T^ with independently and identically distributed sequences ξ_i_, if the random variables ξ_n_ which make up the sequence follow the distribution in Equation (22), then the matrix Φ_s_ is full-rank with a probability arbitrarily close to 1.*

**Proof.**  Assuming that the matrix Φ*_s_* satisfying the conditions above is not full-rank, i.e., some coefficients exist for the *i*-th row in this matrix so that

ξ*_i_* = *a*_1_ξ_1_ + *a*_2_ξ_2_ + … +*a_i_*_−1_ξ_i−1_ + … + *a_M_*ξ*_M_* (23)

Holds and not all of the coefficients *a*_1_, *a*_2_, …, *a_m_* are zeroes.

If we let the stochastic process {*X*(*n*), *n* = 0, 1, …, *N*} represent the row vector *ξ_i_*, then the average function and the variance function are:

(24) $$EX\left(n\right)=(+1)\frac{1-p}{2}+(-1)\frac{1-p}{2}+0\times p=0$$

(25) $$DX\left(n\right)=E{\left[X(n)-EX(n)\right]}^{2}=E{\left[X(n)\right]}^{2}=\frac{1-p}{2}+\frac{1-p}{2}=1-p.$$

If we let the stochastic process {*Y*(*n*), *n* = 0, 1, …, *N*} represent the vector *ξ_i_* = *a*_1_*ξ*_1_ + *a*_2_*ξ*_2_ + … + *a_i_*_−1_*ξ_i_*_−1_ + …+ *a_M_ξ_M_*, then the mean function and the variance function are:

(26) $$EY\left(n\right)=E\left[\sum_{j\in [1,M],j\neq i}a_{j}\xi_{j}(n)\right]=\sum_{j\in [1,M],j\neq i}a_{j}E\xi_{j}(n)=0$$

(27) $$DY\left(n\right)=E{\left[Y(n)-EY(n)\right]}^{2}=E{\left[Y(n)\right]}^{2}=\sum_{j\in [1,M],j\neq i}^{\infty }a_{j}^{2}D\xi_{j}(n)=\sum_{j\in [1,M],j\neq i}^{\infty }a_{j}^{2}\left(1-p\right).$$

Therefore, *X*(*n*) and *Y*(*n*) represent different stochastic processes. For the discrete stochastic process *X*(*n*), the possible numerical values of the stochastic variable are *x*(*i*) ∈ {+1,−1,0}, and the length of the state space *I_X_* is 3*^N^*. For the discrete stochastic process *Y*(*n*), he possible numerical values of the stochastic variable are −*M* + 1 ≤ *y*(*i*) ≤ *M* − 1, *y*(*i*) ∈ *Z* so that length of the state space *I_Y_* is (2*M* − 1)*^N^*.

If we denote by event A that Equation (27) holds, by event B that the coefficients *a*_1_, *a*_2_,…, *a_i_*_−1_, *a_i_*_+1_, …, *a_M_* are not all zeroes and by event C that only one of the coefficients *a*_1_, *a*_2_, …, *a_i_*_−1_, *a_i_*_+1_,…, *a_M_* is zero. Then:

*P*(*A*|*B*) < *P*(*A*|*C*). (28)

The calculation of the probability *P*(*A*|*C*) can be changed into calculating the probability that the stochastic processes *X*_1_(*n*) and *X*_2_(*n*) have the same state. According to the distribution in Equation (28), different states in the state space of the stochastic process *X*(*n*) have different probabilities. Because of the convenience of analyses and without the loss of any generalities, we take *p* = 1/3 in Equation (29). Then:

(29) $$P\left(A|B\right)<P\left(A|C\right)=\frac{1}{3^{N}}<<10^{-3}.$$

This completes the proof. □

For the data reconstruction, we employ the Orthogonal Matching Pursuit (OMP) algorithm. The basic idea of the OMP is to select the element from the complete bank in every iteration and performs the orthogonalization with the method of Gram–Schmidt. Then the sampled data are projected to the space formed by the orthogonal elements so that the components and the remainders of the data are obtained with respect to these orthogonal elements. Then similar ways are adopted for the decomposition of the remainders and these remainders quickly tend smaller as the decomposition is further performed. The optimality of the iteration is guaranteed by the recursive orthogonalization on the set of chosen elements and the iteration number is further reduced. For a *k*-sparse measurement matrix with dimension *N* × *t*, this algorithm could guarantee the successful reconstruction with a rather large probability and the computational complexity is only O(*k* × *n* × *t*). We select the index set Γ*_k_* with *k* elements from the set {1, …, *t*} and further calculate the dimension-*n* residue vector *r_m_* = *y* − *a_k_* according to the dimension-*n* approximate vector *a_k_* of received vector *y.*

Step 1: Initialize the residue vector and set the iteration counter *I* = 0 and index set Γ*_k_* = Φ.

Step 2: Solve the following optimization problem and find the optimal index *λ_i_*. *λ_i_* = arg max*_j_*_= 1, 2, …, *t*_|<*r_i_*_−1_, *φ_j_*>|.

Step 3: Expand the index set and the matrix as Γ*_i_* = Γ*_i_*_−1_∪{*λ_i_*} and Φ*_i_* = [Φ*_i_*_−1_, *φ_λi_*], where Φ_0_ is an empty matrix.

Step 4: Handle the following Least Square (LS) problem *x_i_* = arg min*_x_*||*y* − Φ*_i_x*||_2_.

Step 5: Calculate this new estimated data and residue *y_i_* = Φ*_i_x_i_*, *r_i_* = *y* − *y_i_.*

Step 6: *i* = *i* + 1. Go to Step 2 if *i* < *k.*

Step 7: Reconstruct *X*′ and update the nonzero index set Γ*_k_*. The *λ_j_*-th element of *X*′ is equal to the *j*-th element of *x_i_*.

The algorithm code is shown in Algorithm 1.

**Algorithm 1** The detailed step of the data reconstruction algorithm.**Input:** The measurement matrix Φ with size *N* × *t*, the received vector *y* with dimension *n*, the sparsity level *k* with the ideal data matrix. 
**Output:** The reconstructed data vector *X*′ with dimension *t*. 
1. for *i* = 1:size(*k*,2) 
2.    tem1 = find(*R*1 = *k*(*i*)); 
3.    tem2 = [tem2, tem1]; 
4. end for 
5. for *j* = 1:size(tem2,2) 
6.    *s*(tem2(*j*)).LS = *c*; 
7. end for 
8. for *i* = 1:*N* + 1 
9.    level(*i*) = s(*i*).LS; 
10. end for 
11. level1 = level; 
12. *count* = *ones*(1,*N* + 1); 
13. *s*(*n*+1).D = 1: *N*; 
14. *m* = max(Level1); 
15. for *i* = 1:*m*

16.    index = find(level1= =max(level1)); 
17.    for *p* = 1:size(index,2) 
18.      *u* = *R*(index(*p*)); 
19.      *coun*t(*u*) = *count*(index(*p*))+*count*(*u*); 
20.    end for 
21.    level1(index) = 0; 
22. end for 
23. for *i* = 1:*N*

24. for *j* = 1:*N*

25.    if *count*(*i*) > *M*

26.     *s*(*i*).type = CS; 
27.     CS_NUM = CS_NUM + 1; 
28.     node[*i*][*j*].ID_CS = node[*j*].ID_CS; 
29.    else 
30.    *s*(*i*).type = NO_CS; 
31.    NO_CS_NUM = NO_CS_NUM + 1; 
32.    node[*i*][*j*].ID_NOCS = node[*j*].ID_NOCS; 
33.    end if 
34. end for 
35. end for 34. end for 
36. end for

## 4. Experiment Evaluation and Analysis

For the sake of evaluating the performance of the proposed CS-FCDA scheme, we further compare our proposed CS-FCDA mechanism with the Compressed Sensing (CS) [11], DMOA [12] and MTTA [13] with different evaluation criterions. In order to further evaluate the performance of the proposed CS-FCDA, we have run simulations under the following five different environments. (1) The comparison on the data reconstruction error with various packet loss rate. (2) The comparison on the network lifetime and dead sensor number for the proposed CS-FCDA. (3) The performance comparison on the network running time and the remaining node energy. (4) The performance comparison of the network running time and the dead sensor number with different δ parameter. (5) The change of the success probability with time for the data transmission of the proposed CS-FCDA under different paths. These simulations are run on the Matlab R2014a platform and there are altogether 500 sensor nodes in the network while the data sources are assumed with two-dimensional Gaussian distribution.

In this simulation, we deployed the sensor nodes randomly within a target area which is 300 m × 300 m. The numbers of nodes deployed are 100, 300 and 500, respectively, representing different deployment density. The node energy consumption model in paper [12] is adopted for this paper and the equations for the data transmission and reception energy consumption are listed in (31) and (32).

(30) $$E_{Tx}(L,d)=E_{T-elec}\times L+\varepsilon_{fs}\times L\times d^{2}$$

(31) $$E_{Rx}(L)=E_{R-elec}\times L$$

The configuration of other parameters is showed in Table 1.

For the first time we compare the error ratio of data reconstruction of different schemes with different data loss probabilities. The error data reconstruction ratio is defined as in Equation (32). Then we can compare the network energy efficiency with different node deployment density and clustering.

(32) $$\chi =\frac{\sqrt{\sum {}_{i,j}(x(i,j)-x\prime {\left(x,j\right)}^{2}}}{\sqrt{\sum {}_{i,j}{\left(x(i,j)\right)}^{2}}}$$

**Figure 5:**
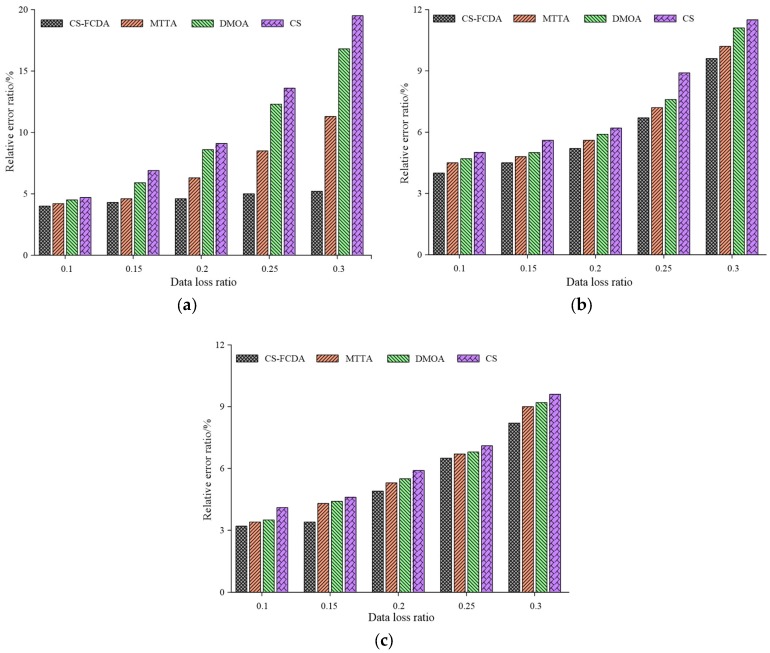
Comparison on relative error ratio with data loss ratio. (**a**) Comparison on relative error ratio with data loss ratio (*N* = 100). (**b**) Comparison on relative error ratio with data loss ratio (*N* = 300). (**c**) Comparison on relative error ratio with data loss ratio (*N* = 500).

The relative error ratio of four different reconstruction schemes with different data loss probabilities are obtained according, as shown in Figure 5a–c respectively. It can be observed that the CS-FCDA scheme exhibits the best performances among the mentioning four schemes. When probability of the data loss increases from 15% to 85%, the data reconstruction error ratio of the proposed CS-FCDA scheme remains between 10% and 30% while the other three schemes exhibit relatively high reconstruction error ratio. For example, the highest reconstruction error ratio of the DMOA scheme is close to 60%. Compared with the results in Figure 5a,b. The comparison among four different schemes is shown in Figure 5c, which shows the relationship between the number of dead nodes and the network lifetime. It is shown in Figure 5c that when the data reconstruction error remains the same, the network running time of the proposed algorithm is relatively high. This is mainly due to the fact that the correlation of the data reconstruction is higher than the other algorithms.

**Figure 6:**
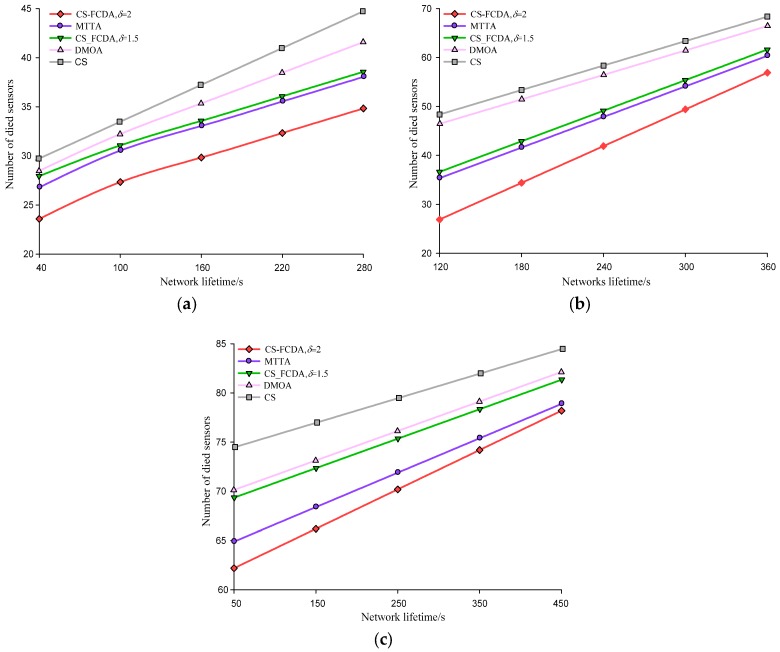
Comparison on network lifetime. (**a**) Comparison on network lifetime when *N* = 100. (**b**) Comparison on network lifetime when *N* = 300. (**c**) Comparison on network lifetime when *N* = 500.

The network lifetime in three density levels of different node is different. The comparison of that is shown in Figure 6a–c. These three different settings represent sparse (*N* = 100), medium (*N* = 300), and dense (*N* = 500) node deployment environment. That the total number of dead sensors increases with the network lifetime can be seen from the figures. Meanwhile, the CS-FCDA scheme improves the network life time by 10.19%, 16.52%, and 23.76% compared with the MTTA, the DMOA and the CS schemes. It is also shown that compared with the sparse deployment environment, the CS-FCDA scheme could obviously prolong the network lifetime when the sensor nodes are densely deployed. In addition, we also compare the residual energy of different reconstruction schemes. In the sparse node deployment environment, when the number of dead nodes is 60, we randomly choose 300 work nodes and compare their residual energy.

**Figure 7:**
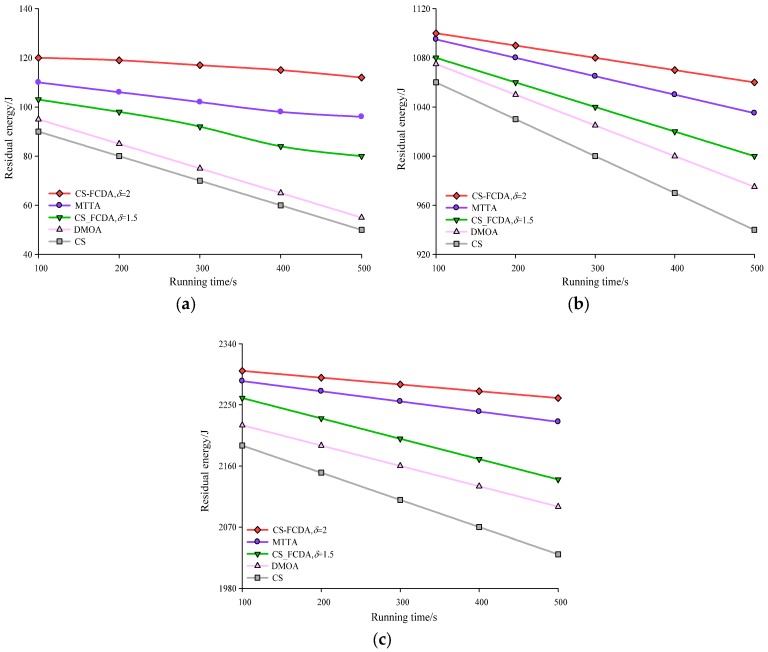
Comparison between network running time and remaining energy. (**a**) Comparison between network running time and remaining energy (*N* = 100). (**b**) Comparison between network running time and remaining energy (*N* = 300). (**c**) Comparison between network running time and remaining energy (*N* = 500).

Figure 7a–c produces the comparison diagrams of the network running time and remaining energy in different amount of nodes. As shown in the Figure 7, with the increasing of the network running time, the energy of the sensing nodes in the four algorithms are all reduced. CS algorithm and DMOA algorithm are reflected in a sort of constant information transmission in the sensing nodes, therefore, the network consumption becomes fast. This paper uses the different value of to control the way of data transmission between the sensing nodes, through the self-adaptive parameter of to make the whole network adjust itself to reach the balance state of energy in the whole network. The comparison results are shown in Figure 7. According to the computation, the proposed CS-FCDA scheme increases the residual energy of the MTTA, DMOA, and CS schemes by 6.63%, 13.17%, and 29.35%, respectively.

**Figure 8:**
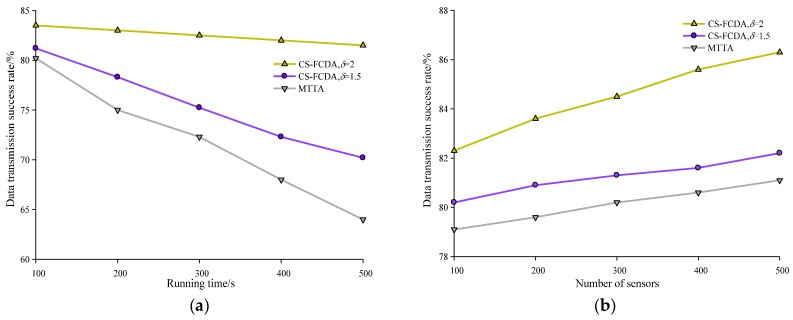
Success probability for data transmission. (**a**) Comparison between network running time and data transmission success rate. (**b**) Comparison between network number of sensors and data transmission success rate.

The success probability of data transmission for the proposed algorithms CS-FCDA and MTTA are illustrated in Figure 8. In Figure 8a, we depict the success probability of data transmission over one single path while the horizontal axis represents the time. In Figure 8b, the success probability for the data transmission is shown while we take different numbers of sensors over multiple data transmission paths. It is shown in Figure 8a that when the number of nodes is *N* = 200, the success probability of data transmission gradually decreases for both algorithms, while the decrease for the MTTA is more prominent. The proposed CS-FCDA could achieve a success probability higher than 82% over the single transmission path with *λ* = 1.5. When *λ* = 2, the success probability over the single transmission path is higher than 87%. According to Figure 8b, when we take different numbers of sensors and employ multiple paths for transmission, the proposed CS-FCDA significantly outperforms the MTTA in terms of the success probability. If the number of paths is larger than 3, the success probability of the proposed CS-FCDA is higher than 86%. This improvement in success probability is mainly due to the modification in the proposed algorithm for the twine problem of multiple disjoint paths.

**Figure 9:**
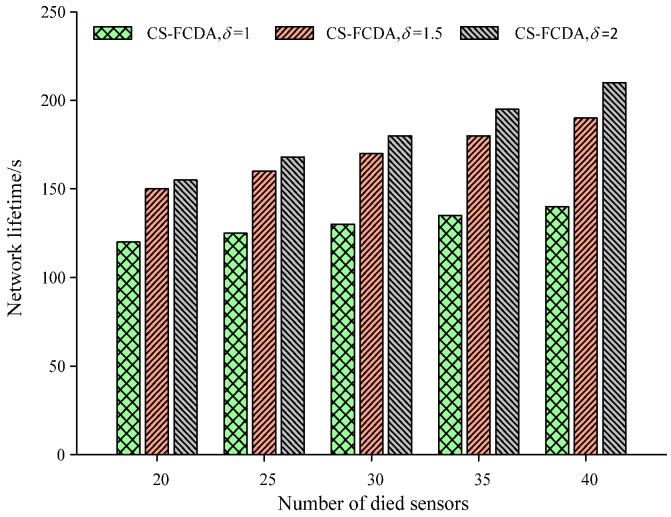
Comparison on network lifetime with different network clustering.

The comparison on network lifetime is shown in Figure 9 with different network clustering. The network is divided into three different numbers of clusters, which correspond to different cluster sizes. It could be easily observed from Figure 9 that when *λ* = 2, the network lifetime can be maximized. If the accurate size of each cluster is indeed close to the node overage area, this indicates that the efficiency of node energy is maximized. The reason is that, in this case, the node could maintain better coverage and network connectivity and the cluster head could communicate most efficiently with cluster members and other cluster heads.

## 5. Conclusions

Due to the limited energy, computation and storage ability of WSNs, we proposed a CS-FCDA scheme. This scheme could exploit the temporal and spatial sparseness feature of the data and further reduce the communication amount among network nodes by efficiently handling the original data. When the data loss probability of the network is relatively high, the proposed scheme could still reconstruct most of the original data and further enhance the Fault-tolerant-correcting capability in the data aggregation process. Meanwhile, the proposed scheme employed the network clustering technology and appropriately divided the network into several clusters. This could optimize and balance the load among all the nodes and improve the communication and energy efficiency of the whole network. Therefore, the network lifetime could be effectively prolonged while guaranteeing the data transmission.

## Figures and Tables

**Table 1:** Parameters of the environment for the experiment.

Parameter	Value	Parameter	Value
radius of sensing (*r_s_*)	10 m	data packet size	80 B
Com-radius	300 m	round time	600 s
sensing angle (α)	π/2	*E_T-elec_*	50 nJ/bit
number of nodes (N)	500	*E_R-elec_*	100 nJ/bit
node energy (E)	5 J	*ε_fs_*	10 pJ/bit/m^2^
